# Elective nodal irradiation versus involved-field irradiation in patients with esophageal cancer receiving neoadjuvant chemoradiotherapy: a network meta-analysis

**DOI:** 10.1186/s13014-019-1388-8

**Published:** 2019-10-16

**Authors:** Tingting Liu, Silu Ding, Jun Dang, Hui Wang, Jun Chen, Guang Li

**Affiliations:** 1 Department of Radiation Oncology, The First Hospital of China Medical University, Shenyang, China; 2 Department of Radiation Oncology, Anshan Cancer Hospital, Anshan, China; 3 Department of Radiation Oncology, General Hospital of Benxi Iron & Steel Industry Group of Liaoning Health Industry Group, Shenyang, China; 4 Department of Radiation Oncology, Shenyang Chest Hospital, Shenyang, China

**Keywords:** Esophagus cancer, Neoadjuvant chemoradiotherapy, Elective nodal irradiation, Involved-field irradiation, Network meta-analysis

## Abstract

**Background:**

To assess the comparative efficacy and safety of elective nodal irradiation (ENI) and involved-field irradiation (IFI) in patients with esophageal cancer (EC) receiving neoadjuvant chemoradiotherapy plus surgery (nCRTS).

**Material and methods:**

PubMed, Embase, Cochrane Library, Web of Science and major meetings were searched for randomized controlled trials (RCTs) that compared at least two of the following treatment regimens: nCRTS, neoadjuvant chemotherapy plus surgery (nCTS), and surgery (S) alone. Overall survival (OS) was the primary outcomes of interest, reported as hazard ratio (HR) and 95% confidence intervals (CIs). A Bayesian network meta-analysis was performed to compare all regimens simultaneously.

**Results:**

Twenty-nine RCTs with a total of 5212 patients were included in the meta-analysis. Both nCRTS adopting ENI (nCRTS-ENI) (HR = 0.63, 95% CI: 0.48–0.83) and nCRTS adopting IFI (nCRTS-IFI) (HR = 0.75, 95% CI: 0.66–0.86) significantly improved OS compared to S alone. No significant differences in OS, locoregional recurrence, distant metastases, R0 resection and postoperative mortality were observed between nCRTS-ENI and nCRTS-IFI. In subgroup analyses, nCRTS-IFI showed a significant OS advantage over nCTS (HR = 0.78, 95% CI: 0.63–0.96) and S alone (HR = 0.50, 95% CI: 0.38–0.68) for esophagus squamous cell carcinoma (ESCC), but nCRTS-ENI did not; nCRTS-ENI using three-dimensional radiotherapy (3D-RT) resulted in an improved OS compared to that with 2D-RT (HR = 0.58, 95% CI: 0.34–0.99). Based on treatment ranking in term of OS, nCRTS-IFI (0.90) and nCRTS-ENI (0.96) was ranked the most effective treatment for ESCC and esophagus adenocarcinoma (EAC), respectively.

**Conclusion:**

Either adopting ENI or IFI, nCRTS is likely to be the optimal treatment for resectable EC, and nCRTS-IFI and nCRTS-ENI seem to be more effective for patients with ESCC and EAC, respectively. Future head to head comparison trials are needed to confirm these findings.

## Introduction

Esophagus cancer (EC) is the eighth most common cancer worldwide and the sixth most common cause of cancer-related deaths [1, 2]. Surgery is still considered as a major component of treatment for all resectable cases. However, surgery alone (S alone) showed poor long-term outcomes, and the 5-year survival rate was rarely > 30% even after curative resection [3, 4]. Some recent randomized control trials (RCTs) have demonstrated the survival benefit of neoadjuvant chemoradiotherapy followed by surgery (nCRTS) compared with S alone [5–8]. While, there are also trials reporting negative results [9–22].

It should be noted that radiation fields used for patients receiving nCRTS are inconsistent in trials, which might affect the outcomes. Some trials adopted elective nodal irradiation (ENI, nodal target volume covering both metastatic lymph nodes and regional nodes) [17–22], and others adopted involved-field irradiation (IFI, nodal target volume including only the metastatic nodes) [5–16]. Efficacy of ENI and IFI has been compared in patients with locally advanced EC undergoing radical CRT in some retrospective studies [23–26], but with different results. At present, no trials have compared the two radiation fields directly in patients undergoing nCRTS, and therefore, there are still questions around which is more superior, and what is the suitable patient population for adopting ENI or IFI.

In light of these issues, we performed a network meta-analysis to assess the comparative effectiveness and safety of ENI and IFI, attempting to identify the best radiation field in patients receiving nCRT.

## Materials and methods

### Literature search strategy

This meta-analysis was conducted in accordance with the Preferred Reporting Items for Systematic Reviews and Meta-analysis (PRISMA) criteria [27] (Additional file 1: Tables S1). PubMed, Embase, Cochrane Library, Web of Science were searched for the available studies published before April 1, 2019, using the strategy as shown in Additional file 1: Tables S2. The reference lists of retrieved studies were manually scanned for relevant additional studies missed by the electronic search.

### Inclusion and exclusion criteria

Studies were included if they met the following criteria: (1) types of studies: RCTs; (2) types of participants: resectable EC; (3) types of interventions: compared at least two of the following treatments: nCRTS, neoadjuvant chemotherapy plus surgery (nCTS), and S alone; and (4) outcomes: overall survival (OS), locoregional recurrence (LR), distant metastases (DM), R0 resection, and postoperative mortality (POM) data. Studies which failed to meet the above criteria were excluded from the network meta-analysis.

### Data extraction

The data were extracted by two investigators independently. The following data were extracted from each study: first author or name of individual RCT, years of publication, duration of the study, country of origin, treatments, numbers of patients, pathologic type, and data of OS, LR, DM, R0 resection, and POM.

### Quality assessment

The methodological quality of RCTs was assessed by Cochrane risk of bias tool [28], which consists of the following five domains: sequence generation, allocation concealment, blinding, incomplete data, and selective reporting. A RCT was finally rated as “low risk of bias” (all key domains indicated as low risk), “high risk of bias” (one or more key domains indicated as high risk), and “unclear risk of bias”.

### Statistical analysis

The primary outcome was OS, and the secondary outcomes were LR, DM, R0 resection, and POM. Hazard ratios (HRs) or odds ratios (ORs) and their 95% confidence intervals (CIs) were used as summary statistics. For direct comparisons, standard pairwise meta-analysis was performed. A statistical test for heterogeneity was performed using the chi-square (*χ*^2^) and *I*-square (*I*^2^) tests with the significance set at *I*^2^ > 50% or *P* < 0.10. If significant heterogeneity existed, a random-effects analysis model was used; otherwise, a fixed-effects model was used.

The Bayesian network-meta analysis (NMA) was performed in a random-effect model using Markov chain Monte Carlo methods [29, 30] in JAGS and the GeMTC package in R (https://drugis.org/software/r-packages/gemtc). For each outcome measure, four independent Markov chains were simultaneously run for 20,000 burn-ins and 100,000 inference iterations per chain to obtain the posterior distribution. The traces plot and Brooks-Gelman-Rubin method were used to assess the convergence of model [31]. Treatment effects were estimated by HR/OR and corresponding 95% CI. Network consistency was assessed with node-split models by statistically testing between direct and indirect estimates within treatment loop [32]. To rank probabilities of all available treatments, the surfaces under the cumulative ranking curve (SUCRAs) were calculated [33]. SUCRA equals one if the treatment is certain to be the best and zero if it’s certain to be the worst [33]. In addition, we conducted subgroup analyses according to histologic type, RT dose, and RT technique. Lastly, comparison-adjusted funnel plot was used to detect the presence of small-study effects or publication bias [34].

## Results

### Literature search results and characteristics of included studies

**Fig. 1:**
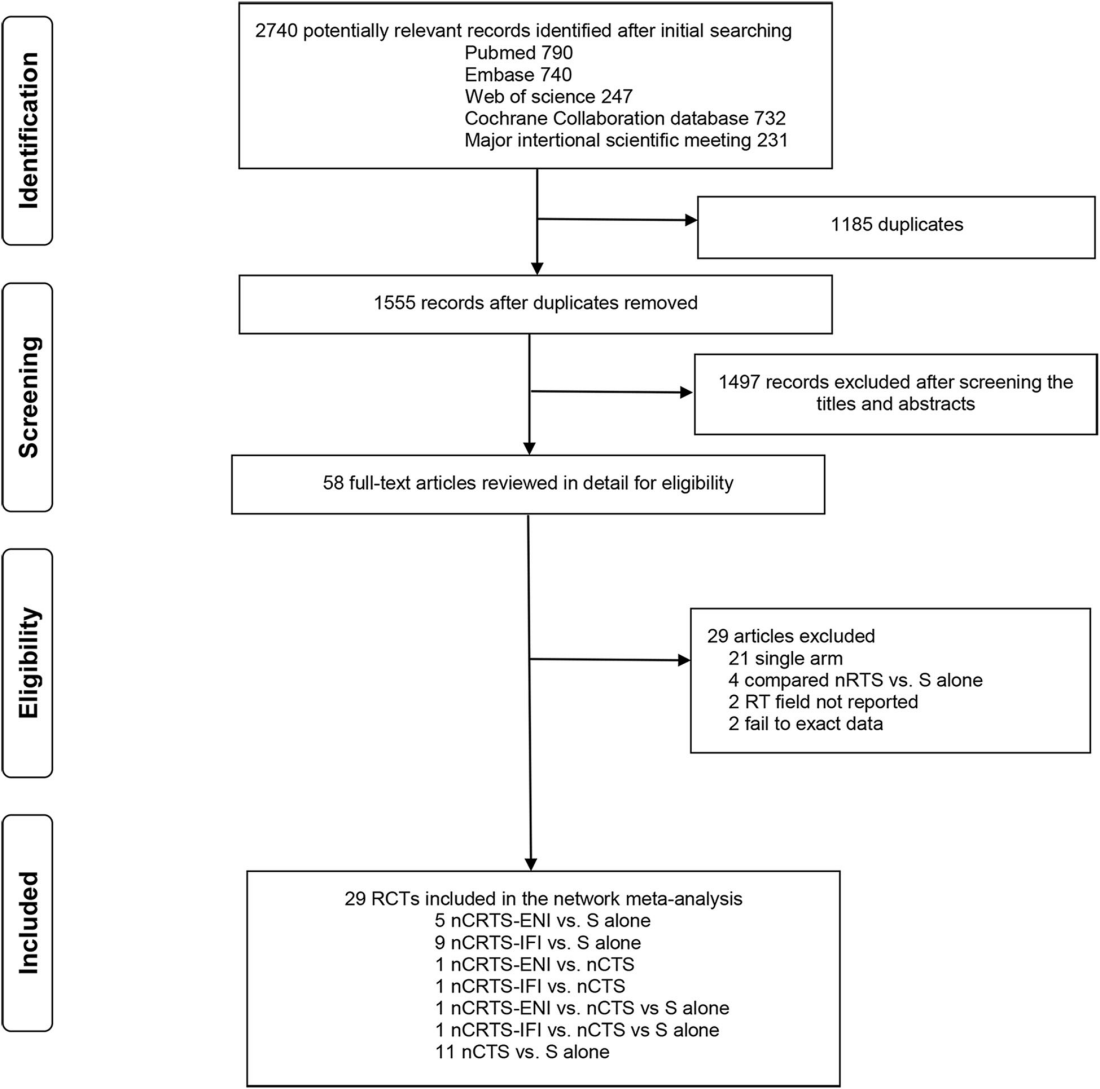
Literature search and selection. RCTs, randomized control trials; nCRTS, neoadjuvant chemoradiotherapy plus surgery; nCTS, neoadjuvant chemotherapy plus surgery; nRTS, neoadjuvant radiotherapy plus surgery; S, surgery; RT, radiotherapy; ENI, elective nodal irradiation; IFI, involved-field irradiation

The literature search results and study selection process are shown in Fig. 1. The initial search retrieved 2740 studies. After removing the duplicates, 1555 citations were identified, and 1497 of them were excluded through an abstract review. The remaining 58 studies were screened through a full-text review for further eligibility. Finally, 29 RCTs [5–22, 35–50] with 5212 patients were included in the meta-analysis. Among them, 5 compared nCRTS using ENI (nCRTS-ENI) with S alone [17–21], 9 compared nCRTS using IFI (nCRTS-IFI) with S alone [5–15], 11 compared nCTS with S alone [38–50], 1 compared nCRTS-ENI and nCTS with S alone [22], 1 compared nCRTS-IFI and nCTS with S alone [16], 1 compared nCRTS-ENI with nCTS [35, 36], and 1 compared nCRTS-IFI with nCTS [37]. The study characteristics are shown in Table 1. Details of radiation fields are shown in Additional file 1: Tables S3.

**Table 1:** Characteristics of included trials

Trial	Time Range	Region	Treatment	Sample size	Median follow-up	Median Age	pStage	Histology	CT	RT	RT
									regimen	dose (Gy)	technique
NEOCRTEC5010/2018 [5]	2007–2014	China	nCRTS-IFI	224	41 m	56	I-IV	SCC	NP	40	3D
			S alone	227		58					
CROSS/2011 [6, 7]	2004–2008	Netherlands	nCRTS-IFI	178	84 m	60	I-III	SCC/AC	PC	41.4	3D
			S alone	188		60					
Lv/2010 [8]	1997–2004	China	nCRTS-IFI	80	45 m	NR	I-III	SCC	PC	40	2D
			S alone	80							
FFCD9901/2014 [9]	2000–2009	France	nCRTS-IFI	98	94 m	58.1	I-III	SCC/AC	FP	45	3D
			S alone	97		57.6					
IG9401/2005 [10]	1994–2000	Australia	nCRTS-IFI	128	65 m	61	NR	SCC/AC	FP	35	2D
			S alone	128		62					
Urba/2001 [11]	1985–1987	America	nCRTS-IFI	50	98 m	62	NR	SCC/AC	FP + Vin	45	3D
			S alone	50		64					
Bosset/1997 [12]	1989–1995	France	nCRTS-IFI	143	55 m	56.6	I-III	SCC	Cis	37	3D
			S alone	139		56.7					
Walsh/1996 [13, 14]	1990–1995	Ireland	nCRTS-IFI	58	10 m	65	I-IV	AC	FP	40	2D
			S alone	55		65					
Apinop/1994 [15]	1986–1992	Thailand	nCRTS-IFI	35	NR	59.6	NR	SCC	FP	40	2D
			S alone	34		59.8					
Cao/2009 [16]	1991–2000	China	nCRTS-IFI	118	NR	NR	II-IV	SCC	FP	40	2D
			nCTS	119							
			S alone	118							
Yanagi/2018 [17]	1997–2001	Japan	nCRTS-ENI	20	90 m	61.5	I-IV	SCC	FP	40	NR
			S alone	21		60					
CALGB9781/2008 [18]	1997–2000	America	nCRTS-ENI	30	72 m	59.9	NR	SCC/AC	FP	50.4	3D
			S alone	26		62.2					
Natsugoe/2006 [19]	1997–2001	Japan	nCRTS-ENI	22	24 m	NR	II-IV	SCC	FP	40	NR
			S alone	23							
Lee/2004 [20]	1999–2002	Korea	nCRTS-ENI	51	25 m	63	I-IV	SCC	FP	45.6	2D
			S alone	50		63					
Le Prise/1994 [21]	1988–1991	France	nCRTS-ENI	41	16 m	56	NR	SCC	FP	20	2D
			S alone	45		59					
Nygaard/1992 [22]	1983–1988	Norway	nCRTS-ENI	53	NR	60.1	NR	SCC	Cis + Ble	35	2D
			nCTS	56		62.9					
			S alone	50		61.4					
Stahl/2009 [35, 36]	2000–2005	Germany	nCRTS-ENI	60	126 m	60.6	I-IV	AC	PLF	30	3D
			nCTS	59		56					
Burmeister/2011 [37]	2000–2006	Australia	nCRTS-IFI	39	94 m	60	I-III	AC	FP	35	3D
			nCTS	36		63					
Boonstra/2011 [38]	1989–1996	Netherlands	nCTS	85	15 m	60	I-IV	SCC	EP		
			S alone	84	14 m	60					
Ychou/2011 [39]	1995–2003	Multicenter	nCTS	84	NR	NR	NR	AC	FP		
			S alone	85							
OEO2/2002 [40, 41]	1992–1998	UK	nCTS	400	73 m	63	NR	SCC/AC	FP		
			S alone	402		63					
MAGIC/2006 [42]	1994–2002	Multicenter	nCTS	65	NR	NR	NR	AC	ECF		
			S alone	66							
RTOG8911/2007 [43, 44]	1990–1995	Multicenter	nCTS	233	NR	61	NR	SCC/AC	FP		
			S alone	234		62					
Ancona/2001 [45]	1992–1997	Italy	nCTS	47	NR	58	NR	NR	FP		
			S alone	47		58					
Baba/2000 [46]	1993–1995	Japan	nCTS	21	NR	63.6	I-IV	SCC	PLF		
			S alone	21		60.1					
Law/1997 [47]	1989–1995	China	nCTS	74	17 m	64	I-III	SCC	FP		
			S alone	73		63					
Schlag/1992 [48]	NR	Germany	nCTS	35	8 m	NR	NR	SCC	FP		
			S alone	42							
Maipang/1994 [49]	1988–1990	Thailand	nCTS	24	NR	64.2	NR	SCC	Cis + Ble		
			S alone	22		64.8					
Roth/1988 [50]	1982–1986	America	nCTS	19	30 m	NR	NR	NR	NP + Ble		
			S alone	20							

*Abbreviations*: *m* Months, *UK* United Kingdom, *nCRTS* Neoadjuvant chemoradiotherapy plus surgery, *nCTS* Neoadjuvant chemotherapy plus surgery, *S* Surgery, *CT* Chemotherapy, *RT* Radiotherapy, *ENI* Elective nodal irradiation, *IFI* Involved-field irradiation, *Cis* Cisplatin, *Vin* Vinblastine, *FP* Fluorouracil/cis, *PC* Paclitaxel/cis, *NP* Vinorelbine/cis, *PLF* Fluorouracil/leucovorin/cis, *Ble* Bleomycin, *ECF* Epirubicin/cisplatin/fluorouracil, *SCC* Squamous cell carcinoma, *AC* Adenocarcinoma, *2D* Two-dimensional RT, *3D* Three-dimensional RT, *NR* Not reported

### Assessment of included trial

The risk of bias in included RCTs was summarized in Additional file 1: Figure S1. Seven trials [13–16, 21, 22, 48, 49] were judged to be unclear risk of bias, as they had more than three domains indicating as unclear risk. The remaining trials were rated with a low risk of bias. Funnel plot analysis in term of OS did not indicate any evident risk of publication bias (Additional file 1: Figure S2).

### Conventional pairwise meta-analysis

Results of direct comparison meta-analysis are shown in Table 2. nCRTS-ENI (HR = 0.70, 95% CI: 0.54–0.92, *I*^*2*^ = 8%), nCRTS-IFI (HR = 0.74, 95% CI: 0.66–0.83, *I*^*2*^ = 10%), and nCTS (HR = 0.86, 95% CI: 0.76–0.98, *I*^*2*^ = 40%) showed significant OS advantage over S alone. Compared to S alone, nCRTS-IFI and nCTS showed a significant decrease in LR (OR = 0.43, 95% CI: 0.33–0.57, *I*^*2*^ = 0% and OR = 0.79, 95% CI: 0.62–0.99, *I*^*2*^ = 26%), and a trend of decrease in DM (OR = 0.79, 95% CI: 0.62–1.00, *I*^*2*^ = 0% and OR = 0.83, 95% CI: 0.68–1.01, *I*^*2*^ = 37%). nCRTS-ENI (OR = 5.75, 95% CI: 2.19–15.13, *I*^*2*^ = 0%), nCRTS-IFI (OR = 5.17, 95% CI: 1.95–13.67, *I*^*2*^ = 68%), and nCTS (OR = 1.71, 95% CI: 1.39–2.10, *I*^*2*^ = 0%) significantly increased R0 resection compared to S alone. nCRTS-ENI also increased R0 resection than nCTS (OR = 4.71, 95% CI: 1.98–11.24, *I*^*2*^ = 0%). nCRTS-IFI resulted in a significantly higher POM than S alone (OR = 1.79, 95% CI: 1.14–2.82, *I*^*2*^ = 27%).

**Table 2:** Results of direct comparsions

Outcome	Treatment	No. of studies	No. of patients	HR/OR(95%CI)	Heterogeneity	
					I^2^(%)	*P*
OS	nCRTS-ENI vs S alone	6	432	**HR 0.70(0.54–0.92)**	8	0.37
	nCRTS-IFI vs S alone	10	2228	**HR 0.74(0.66–0.83)**	10	0.35
	nCTS vs S alone	13	2526	**HR 0.86(0.76–0.98)**	40	0.06
LR	nCRTS-ENI vs S alone	4	288	OR 0.69(0.35–1.35)	46	0.13
	nCRTS-IFI vs S alone	6	1221	**OR 0.43(0.33–0.57)**	0	0.50
	nCTS vs S alone	7	2176	**OR 0.79(0.62–0.99)**	26	0.23
DM	nCRTS-ENI vs S alone	4	288	OR 0.87(0.35–2.21)	57	0.07
	nCRTS-IFI vs S alone	6	1221	**OR 0.79(0.62–1.00)**	0	0.43
	nCTS vs S alone	7	2176	**OR 0.83(0.68–1.01)**	37	0.15
R0 resection	nCRTS-ENI vs S alone	2	155	**OR 5.75(2.19–15.13)**	0	0.61
	nCRTS-IFI vs S alone	4	1119	**OR 5.17(1.95–13.67)**	68	0.02
	nCTS vs S alone	7	1705	**OR 1.71(1.39–2.10)**	0	0.75
	nCRTS-ENI vs nCT	2	166	**OR 4.71(1.98–11.24)**	0	0.85
POM	nCRTS-ENI vs S alone	5	324	OR 1.52(0.66–3.52)	0	0.85
	nCRTS-IFI vs S alone	8	1704	**OR 1.79(1.14–2.82)**	27	0.21
	nCTS vs S alone	11	2453	OR 1.02(0.75–1.38)	0	0.87

*Abbreviations*: *No.* Number, *HR* Hazard ratio, *CI* Confidence interval, *OR* Odds ratio, *OS* Overall survival, *LR* Locoregional recurrence, *DM* Distant metastases, *POM* Post-operative mortality, *nCRTS* Neoadjuvant chemoradiotherapy plus surgery, *nCTS* Neoadjuvant chemotherapy plus surgery, *S* Surgery, *ENI* Elective nodal irradiation, *IFI* Involved-field irradiation

Significant results are in bold

### Network meta-analysis

**Fig. 2:**
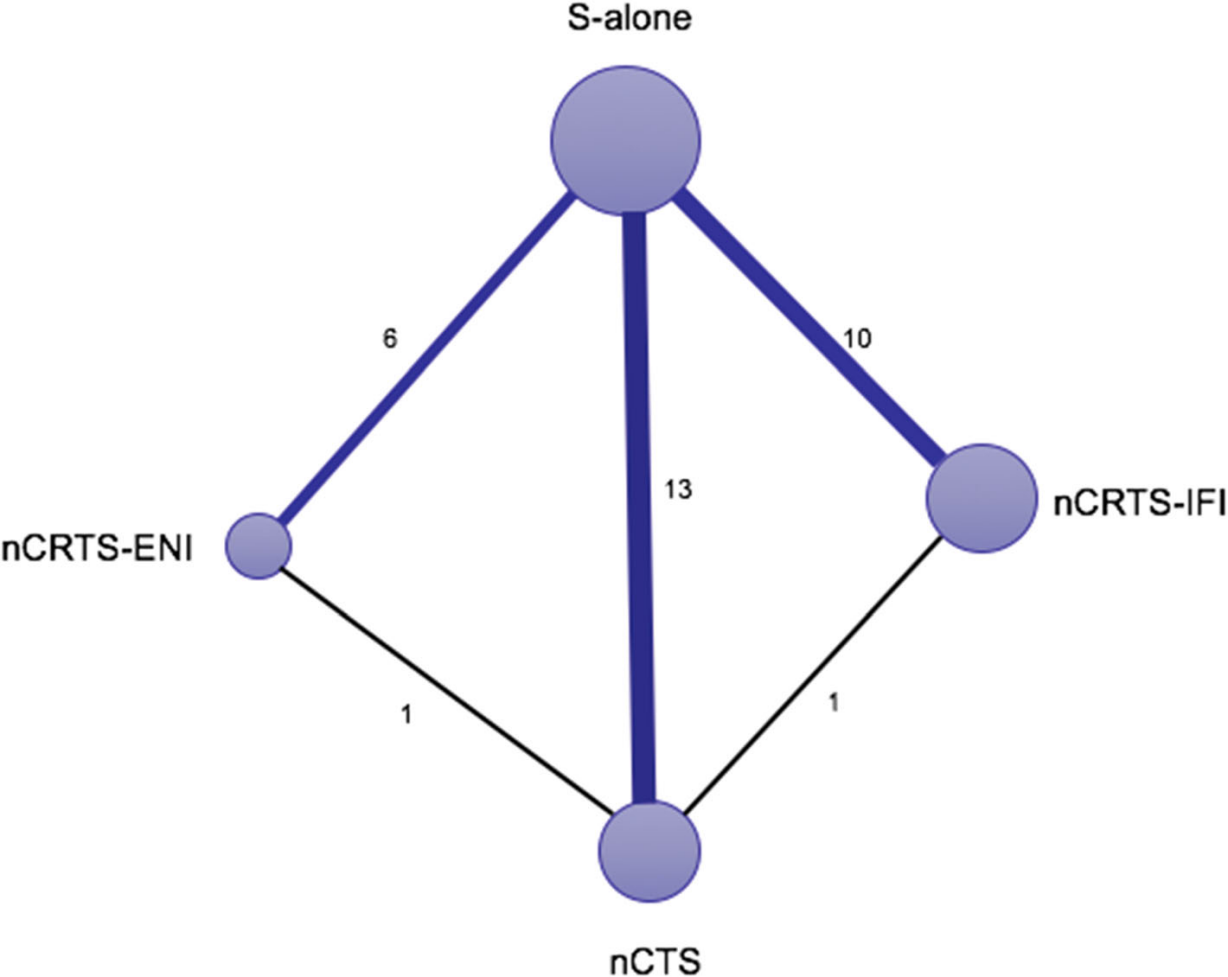
Network of eligible comparisons for the Bayesian network meta-analysis. The size of the nodes is proportional to the number of patients (in parentheses) randomized to receive the treatment. The width of the lines is proportional to the number of trials (beside the line) comparing the connected treatments. nCRTS, neoadjuvant chemoradiotherapy plus surgery; nCTS, neoadjuvant chemotherapy plus surgery; S, surgery; ENI, elective nodal irradiation; IFI, involved-field irradiation

Figure 2 shows the network plot established for NMA for OS. Results of the NMA are presented in Table 3a. nCRTS-ENI (HR = 0.63, 95% CI: 0.48–0.83, *P* = 0.001), nCRTS-IFI (HR = 0.75, 95% CI: 0.66–0.86, *P* < 0.001), and nCTS (HR = 0.87, 95% CI: 0.77–0.97, *P* = 0.012) significantly improved OS compared to S alone; nCRTS-ENI also showed a significant OS advantage over nCTS (HR = 0.73, 95% CI: 0.55–0.97, *P* = 0.03). nCRTS-IFI significantly decreased LR compared to nCTS (OR = 0.59, 95% CI: 0.37–0.94, *P* = 0.03) and S alone (OR = 0.43, 95% CI: 0.30–0.60, *P* < 0.001). S alone and nCTS showed a lower R0 resection than nCRTS-ENI (OR = 0.16, 95% CI: 0.07–0.34, *P* < 0.001 and OR = 0.29, 95% CI: 0.13–0.59, *P* < 0.001) and nCRTS-IFI (OR = 0.16, 95% CI: 0.09–0.28, *P* < 0.001 and OR = 0.28, 95% CI: 0.14–0.53, *P* < 0.001). S alone had a lower POM than nCRTS-IFI (OR = 0.56, 95% CI: 0.33–0.92, *P* = 0.02). No significant difference in OS, LR, DM, R0 resection, and POM were observed between nCRTS-ENI and nCRTS-IFI.

**Table 3:** Network meta-analysis results

a. Network meta-analysis results for five outcomes					
OS					
nCRTS-ENI					
0.84(0.62–1.1)	nCRTS-IFI				
**0.73(0.55–0.97)**	0.87(0.73–1.0)	nCTS			
**0.63(0.48–0.83)**	**0.75(0.66–0.86)**	**0.87(0.77–0.97)**	S-alone		
LR					
nCRTS-IFI					
0.74(0.37–1.5)	nCRTS-ENI				
**0.59(0.37–0.94)**	0.61(0.30–1.3)	nCTS			
**0.43(0.30–0.60)**	0.58(0.31–1.1)	0.79(0.59–1.1)	S-alone		
DM					
nCRTS-IFI					
1.0(0.54–1.9)	nCRTS-ENI				
0.92(0.60–1.4)	0.90(0.50–1.6)	nCTS			
0.79(0.57–1.1)	0.76(0.44–1.3)	0.85(0.64–1.2)	S-alone		
POM					
S-alone					
0.99(0.68–1.4)	nCTS				
**0.56(0.33–0.92)**	0.56(0.30–1.0)	nCRTS-IFI			
0.56(0.27–1.1)	0.56(0.27–1.2)	1.0(0.41–2.4)	nCRTS-ENI		
R0 resection					
S-alone					
**0.57(0.40–0.80)**	nCTS				
**0.16(0.09–0.28)**	**0.28(0.14–0.53)**	nCRTS-IFI			
**0.16(0.07–0.34)**	**0.29(0.13–0.59)**	1.0(0.39–2.6)	nCRTS-ENI		
**b.** Network meta-analysis results of OS for four subgroups					
ESCC					
nCRTS-IFI					
0.83(0.47–1.5)	nCRTS-ENI				
**0.78(0.63–0.96)**	0.80(0.43–1.5)	nCTS			
**0.50(0.38–0.68)**	0.61(0.35–1.0)	0.76(0.57–1.0)	S-alone		
EAC					
nCRTS-ENI					
0.70(0.37–1.3)	nCRTS-IFI				
0.65(0.38–1.1)	0.93(0.71–1.3)	nCTS			
**0.50(0.28–0.87)**	**0.72(0.58–0.91)**	**0.78(0.62–0.93)**	S-alone		
RT with dose of ≥40Gy/<40Gy					
nCRTS-ENI ≥ 40Gy					
0.90(0.59–1.4)	nCRTS-IFI ≥ 40Gy				
0.89(0.54–1.5)	0.99(0.70–1.4)	nCRTS-ENI < 40Gy			
0.71(0.48–1.1)	0.79(0.65–0.96)	0.80(0.58–1.1)	nCTS		
0.68(0.43–1.1)	0.76(0.56–1.0)	0.76(0.51–1.1)	0.96(0.72–1.3)	nCRTS-IFI < 40Gy	
**0.62(0.43–0.92)**	**0.70(0.59–0.82)**	**0.70(0.51–0.96)**	**0.88(0.78–0.99)**	0.92(0.71–1.2)	S-alone
RT with technique of 3DRT/2DRT					
nCRTS-ENI-3DRT					
0.74(0.46–1.2)	nCRTS-IFI-2DRT				
0.68(0.42–1.1)	0.92(0.68–1.2)	nCRTS-IFI-3DRT			
**0.58(0.34–0.99)**	0.87(0.57–1.3)	0.94(0.63–1.4)	nCRTS-ENI-2DRT		
**0.61(0.39–0.94)**	0.83(0.64–1.1)	0.90(0.72–1.1)	0.96(0.66–1.4)	nCTS	
**0.53(0.34–0.80)**	**0.72(0.57–0.88)**	**0.78(0.64–0.94)**	0.82(0.58–1.2)	**0.86(0.76–0.98)**	S-alone

*Abbreviations*: *OS* Overall survival, *LR* Locoregional recurrence, *DM* Distant metastases, *POM* Post-operative mortality, *nCRTS* Neoadjuvant chemoradiotherapy plus surgery, *nCTS* Neoadjuvant chemotherapy plus surgery, *S* Surgery, *RT* Radiotherapy, *ENI* Elective nodal irradiation, *IFI* Involved-field irradiation, *ESCC* Esophagus squamous cell carcinoma, *EAC* Esophagus adenocarcinoma, *2D* Two-dimensional, *3D* Three-dimensional

Significant results are in bold

### Inconsistency assessment and treatment ranking

There were two independent closed loops in the network for OS, LR, DM, and R0 resection: nCRTS-ENI/nCTS/S alone and nCRTS-IFI/nCTS/S alone; one independent closed loop for POM: nCRTS-ENI/nCTS/S alone. Analysis of inconsistency showed that the NMA results were similar to the PWMA results for the five outcomes, which suggested the consistency between the direct and indirect evidence (Additional file 1: Figure S3).

Results of the treatment rankings based on SUCRA are shown in Table 4a. In term of OS, nCRTS-ENI (0.93) was ranked the most effective treatment in term of OS, followed by nCRTS-IFI (0.71). nCRTS-IFI (0.95) was ranked the most effective treatment in term of LR, followed by nCRTS-ENI (0.62). With regard to DM, POM, and R0 resection, SUCRA values were similar between nCRTS-ENI and nCRTS-IFI.

**Table 4:** SUCRA values

a. SUCRA values for five outcomes									
OS		LR		DM		POM		R0 resection	
Treatment	SUCRA	Treatment	SUCRA	Treatment	SUCRA	Treatment	SUCRA	Treatment	SUCRA
nCRTS-ENI	0.93	nCRTS-IFI	0.95	nCRTS-IFI	0.69	S alone	0.83	S alone	1.00
nCRTS-IFI	0.71	nCRTS-ENI	0.62	nCRTS-ENI	0.67	nCTS	0.79	nCTS	0.67
nCTS	0.36	nCTS	0.39	nCTS	0.53	nCRTS-IFI	0.20	nCRTS-IFI	0.19
S alone	0.00	S alone	0.04	S alone	0.11	nCRTS-ENI	0.19	nCRTS-ENI	0.15
b. SUCRA values of OS for four subgroups									
ESCC		EAC		RT dose		RT-technique			
Treatment	SUCRA	Treatment	SUCRA	Treatment	SUCRA	Treatment	SUCRA		
nCRTS-IFI	0.90	nCRTS-ENI	0.96	nCRTS-ENI- ≥ 40Gy	0.86	nCRTS-ENI-3DRT	0.98		
nCRTS-ENI	0.68	nCRTS-IFI	0.63	nCRTS-IFI- ≥ 40Gy	0.75	nCRTS-IFI-3DRT	0.69		
nCTS	0.34	nCTS	0.41	nCRTS-ENI- < 40Gy	0.73	nCRTS-IFI-2DRT	0.54		
S alone	0.08	S alone	0.00	nCTS	0.35	nCRTS-ENI-2DRT	0.42		
				nCRTS-IFI- < 40Gy	0.25	nCTS	0.34		
				S alone	0.05	S alone	0.03		

*Abbreviations*: *SUCRA* Surface under the cumulative ranking curve, *OS* Overall survival, *LR* Locoregional recurrence, *DM* Distant metastases, *POM* Post-operative mortality, *nCRTS* Neoadjuvant chemoradiotherapy plus surgery, *nCTS* Neoadjuvant chemotherapy plus surgery, *S* Surgery, *RT* Radiotherapy, *ENI* Elective nodal irradiation, *IFI* Involved-field irradiation, *ESCC* Esophagus squamous cell carcinoma, *EAC* Esophagus adenocarcinoma, *2D* Two-dimensional, *3D* Three-dimensional

### Subgroup analyses

NMA results of subgroup analyses are shown in Table 3b (SUCRA values are shown in Table 4b). Subgroup analyses for esophagus squamous cell carcinoma (ESCC) and esophagus adenocarcinoma (EAC) were conducted in 23 trials with 3164 patients and 11 trials with 1997 patients, respectively. With regard to ESCC, nCRTS-IFI showed significant OS advantage over S alone and a trend OS advantage over nCTS, and was ranked the most effective treatment (0.90); nCRTS-ENI had a trend OS benefit over S alone. As for EAC, both nCRTS-ENI and nCRTS-IFI significantly improved OS compared to S alone, and nCRTS-ENI was ranked the best treatment (0.96).

In subgroup analysis according to RT dose (18 trials with 2860 patients), nCRTS-IFI with dose of ≥40Gy significantly improved OS compared to S alone, while nCRTS-IFI with dose of <40Gy did not; both nCRTS-ENI with dose of ≥40Gy and < 40Gy showed a significant OS advantage over S alone; and nCRTS-ENI with dose of ≥40Gy was ranked the most effective regimen (0.86).

In subgroup analysis according to RT technique (16 trials with 2774 patients), nCRTS-ENI adopting three-dimensional radiotherapy (3D-RT) significantly improved OS compared to nCRTS-ENI adopting 2D-RT, nCTS, and S alone, and was ranked the most effective regimen (0.99); nCRTS-IFI was more effective than S alone regardless RT technique adopted.

## Discussion

Currently, nCRTS has been the most common treatment approach for patients with resectable EC, but the optimal radiation field remains unidentified. EC is characterized as an aggressive disease, and lymph node metastasis, particularly regional lymph node involvement, usually occurs early. Taking into consideration microscopic spread, some trials adopted ENI instead of IFI for patients receiving nCRTS. In CALGB 9781 trials [18], nCRTS adopting ENI followed by surgery showed a long-term survival advantage over S alone for patients with EC. Nevertheless, there are also trials of a series of cases treated with IFI. Recently, two large phase III trials [5–7] also showed that nCRTS improved survival over surgery alone among patients with esophageal or junctional cancer, while IFI was adopted in RT. To date, there are still no trials that have compared efficacy of the two radiation fields directly in EC patients receiving nCRTS, and which is more effective remains unclear.

To our knowledge, this is the first network meta-analysis assessing the comparative efficacy and safety of nCRTS-ENI and nCRTS-IFI for patients with EC. It showed that both nCRTS-ENI and nCRTS-IFI significantly improved OS compared to S alone. nCRTS-ENI also showed significant OS advantage over nCTS. No significant difference in OS, LR, DM, and POM was observed between nCRTS-ENI and nCRTS-IFI. Based on treatment ranking in term of OS, nCRTS-ENI had the highest probability of being the most effective treatment (93%), followed by nCRTS-IFI (71%) and nCTS (36%).

However, in subgroup analysis according to pathologic type, nCRTS-IFI (90%) was ranked the most effective treatment for ESCC, followed by nCRTS-ENI (68%). nCRTS-IFI showed significant and a trend OS advantage over S alone and nCTS, respectively. While nCRTS-ENI only had a trend OS benefit compared to S alone. In the CROSS trial [6, 7], nCRTS-IFI resulted in improved OS for both ESCC and EAC, but the magnitude of this benefit was greater for ESCC patients (HR for ESCC vs. EAC were 0.48 vs. 0.73 respectively). These results suggested that nCRTS-IFI seemed to be more effective than nCRTS-ENI for patients with ESCC. Future head to head comparison trials are needed to confirm this finding and explore the mechanism.

RT dose and technique used in individual trials were various, which might also affect the outcomes. In our NMA, although nCRTS-ENI and nCRTS-IFI with dose of ≥40Gy seemed to be superior to those with dose of <40Gy based on treatment ranking, there were no significant difference in OS between the two dose group. Moreover, common dose in subgroup of ≥40Gy was only 40–41.4Gy. With developments in RT technique, whether a rather higher dose might be more reasonable needs further investigation.

In subgroup analysis of RT technique, we found that nCRTS-ENI adopting 3D-RT had a significant OS benefit compared to nCRTS-ENI adopting 2D-RT. Compared with 2D-RT, 3D-RT delivered a high dose to the tumor target volume while potentially minimizing the dose to the organ at risk. The results suggested that 3D-RT was more important for EC patients receiving nCRTS-ENI.

Treatment-related toxicities between ENI and IFI have been compared for EC patients receiving radical CRT in several retrospective studies. Results of two small meta-analysis [51, 52] showed that the incidences of esophageal and lung toxicities were significantly higher in ENI group. However, most of trials comparing nCRTS with S alone did not reported CRT-related toxicities in detail, and therefore, indirect comparison of CRT-related toxicities between nCRTS-ENI and nCRTS-IFI could not be performed. In our NMA, nCRTS seemed to had a higher POM than S alone, but no significant difference was observed between nCRTS-ENI and nCRTS-IFI.

There are several limitations in our meta-analysis. Firstly, in common with other meta-analyses, data were collected and analyzed in aggregate on the basis of results reported from trials, instead of individual patient data. Secondly, different operative techniques and CT regimens were adopted in individual trials, which might lead to heterogeneity. Thirdly, most of the studies included patients with mixed stage and tumor location and could not be extracted separately, subgroup analyses according to stage and tumor location could not be performed. Finally, majority of trials comparing nCRTS with surgery alone did not reported RT related toxicities. Thus, the comparison of RT related toxicities between nCRTS-ENI and nCRTS-IFI could not be performed.

## Conclusions

Either adopting ENI or IFI, nCRTS is likely to be the optimal treatment for resectable EC, and nCRTS-IFI and nCRTS-ENI seem to be more effective for patients with ESCC and EAC, respectively. 3D-RT seems to be more important for patients receiving nCRTS-ENI. nCRTS with RT dose of ≥40Gy seems to be superior to that with radiation dose of <40Gy, while the optimal dose remains unclear. Future head to head comparison trials are needed to confirm these findings.

## Supplementary information

**Additional file 1: Figure S1.** Assessment of risk of bias. A: Methodological quality graph: authors’ judgment about each methodological quality item presented as percentages across all included studies; B: Methodological quality summary: authors’ judgment about each methodological quality item for each included study, “+” low risk of bias; “?” unclear risk of bias; “-” high risk of bias. **Figure S2.** Comparison-adjusted funnel plots of publication bias test for overall survival. nCRTS, neoadjuvant chemoradiotherapy plus surgery; nCTS, neoadjuvant chemotherapy plus surgery; S, surgery; ENI, elective nodal irradiation; IFI, involved-field irradiation. **Figure S3.** Inconsistency evaluation by node-splitting analyses. (a) overall survival; (b) locoregional recurrence; (c) distant metastases; (d) R0 resection; (e) post-operative mortality. nCRTS, neoadjuvant chemoradiotherapy plus surgery; nCTS, neoadjuvant chemotherapy plus surgery; S, surgery; ENI, elective nodal irradiation; IFI, involved-field irradiation. **Table S1.** PRISMA NMA Checklist. **Table S2.** Search strategy. **Table S3.** Details of radiation fields.

## Data Availability

Not applicable.

## Abbreviations

3D-RT: Three-dimensional radiotherapy
Cis: Confidence intervals
DM: Distant metastases
EAC: Esophagus adenocarcinoma
EC: Esophagus cancer
ENI: Elective nodal irradiation
ESCC: Esophagus squamous cell carcinoma
HRs: Hazard ratios
IFI: Involved-field irradiation
LR: locoregional recurrence
nCRTS: Neoadjuvant chemoradiotherapy followed by surgery
nCRTS-ENI: nCRTs using ENI
nCRTS-IFI: nCRTS using IFI
nCTS: Neoadjuvant chemotherapy plus surgery
NMA: Network-meta analysis
ORs: Odds ratios
OS: Overall survival
POM: Postoperative mortality
PWMA: Pairwise meta-analysis
RCTs: Randomized control trials
S alone: Surgery alone
SUCRA: Surfaces under the cumulative ranking curve
