# Supplementary figures and images for: Elective nodal irradiation versus involved-field irradiation in patients with esophageal cancer receiving neoadjuvant chemoradiotherapy: a network meta-analysis
Source: Radiat Oncol. 2019 Oct 16;14:176. doi: 10.1186/s13014-019-1388-8 (PMC6794743; doi:10.1186/s13014-019-1388-8)

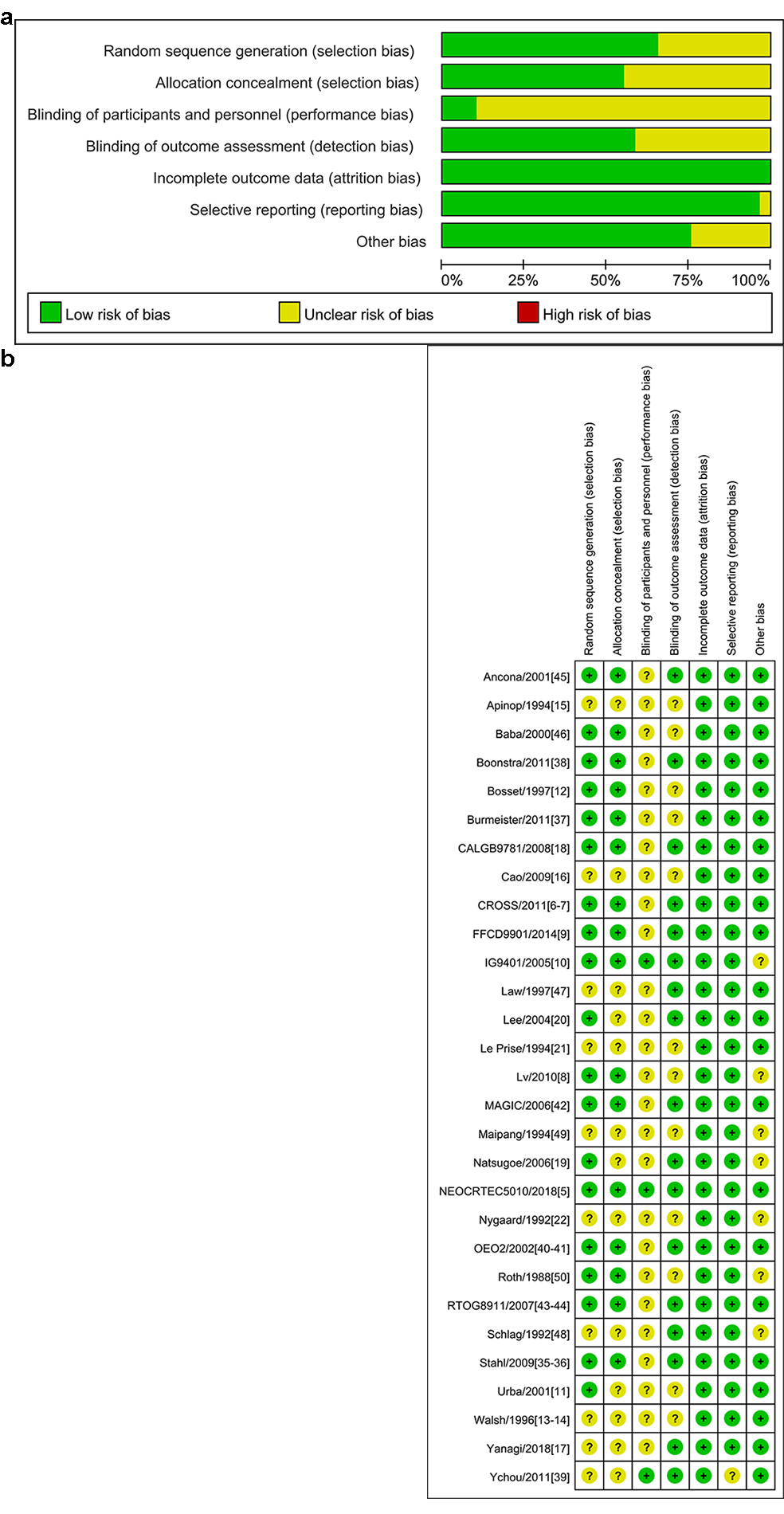

Supplement: Supplementary file 1 — Additional file 1: Figure S1. Assessment of risk of bias. A: Methodological quality graph: authors’ judgment about each methodological quality item presented as percentages across all included studies; B: Methodological quality summary: authors’ judgment about each methodological quality item for each included study, “+” low risk of bias; “?” unclear risk of bias; “-” high risk of bias. Figure S2. Comparison-adjusted funnel plots of publication bias test for overall survival. nCRTS, neoadjuvant chemoradiotherapy plus surgery; nCTS, neoadjuvant chemotherapy plus surgery; S, surgery; ENI, elective nodal irradiation; IFI, involved-field irradiation. Figure S3. Inconsistency evaluation by node-splitting analyses. (a) overall survival; (b) locoregional recurrence; (c) distant metastases; (d) R0 resection; (e) post-operative mortality. nCRTS, neoadjuvant chemoradiotherapy plus surgery; nCTS, neoadjuvant chemotherapy plus surgery; S, surgery; ENI, elective nodal irradiation; IFI, involved-field irradiation. Table S1. PRISMA NMA Checklist. Table S2. Search strategy. Table S3. Details of radiation fields. [file 13014_2019_1388_MOESM1_ESM.zip › Additional file 1 Fig. S1.tiff]

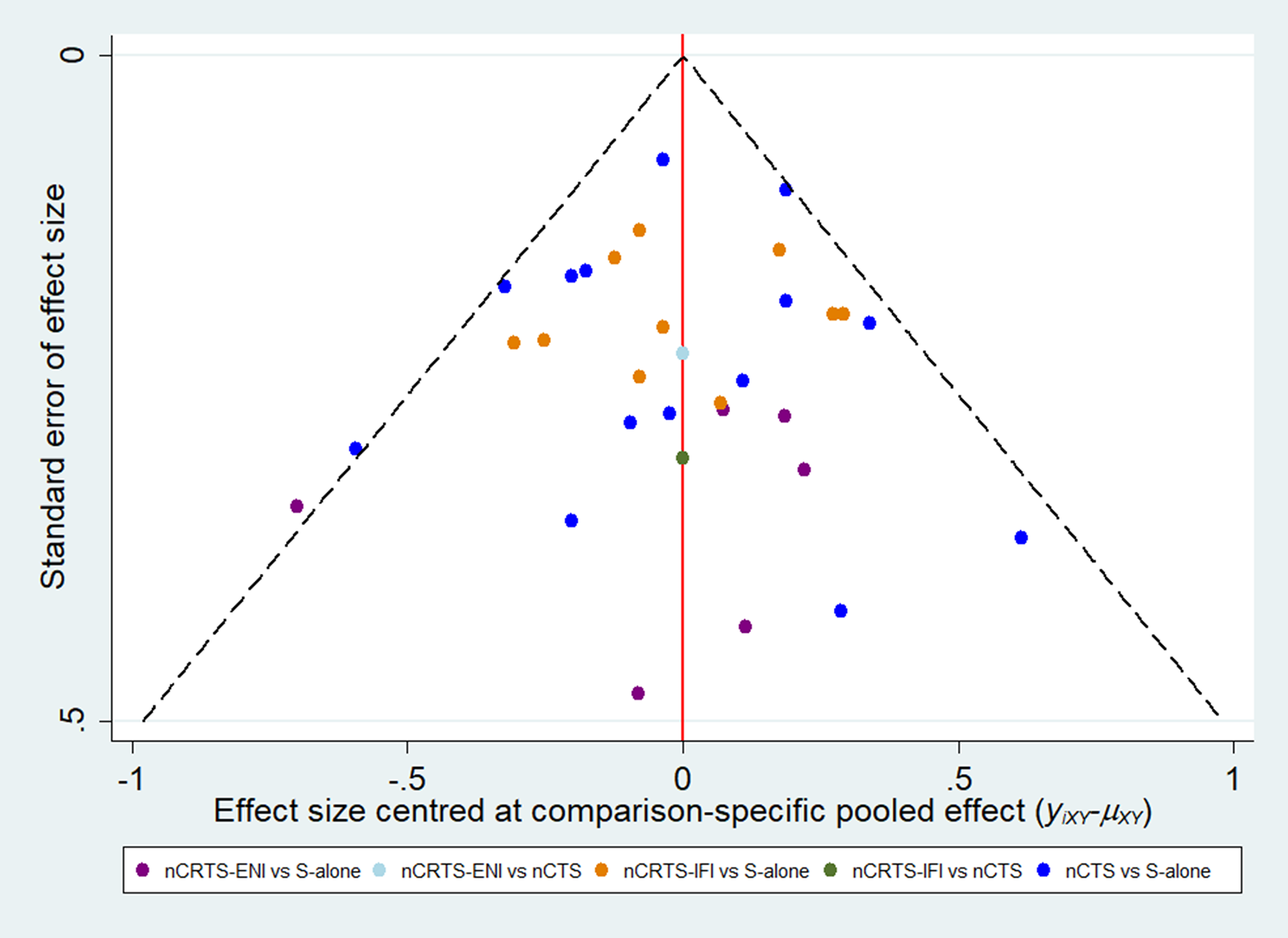

Supplement: Supplementary file 1 — Additional file 1: Figure S1. Assessment of risk of bias. A: Methodological quality graph: authors’ judgment about each methodological quality item presented as percentages across all included studies; B: Methodological quality summary: authors’ judgment about each methodological quality item for each included study, “+” low risk of bias; “?” unclear risk of bias; “-” high risk of bias. Figure S2. Comparison-adjusted funnel plots of publication bias test for overall survival. nCRTS, neoadjuvant chemoradiotherapy plus surgery; nCTS, neoadjuvant chemotherapy plus surgery; S, surgery; ENI, elective nodal irradiation; IFI, involved-field irradiation. Figure S3. Inconsistency evaluation by node-splitting analyses. (a) overall survival; (b) locoregional recurrence; (c) distant metastases; (d) R0 resection; (e) post-operative mortality. nCRTS, neoadjuvant chemoradiotherapy plus surgery; nCTS, neoadjuvant chemotherapy plus surgery; S, surgery; ENI, elective nodal irradiation; IFI, involved-field irradiation. Table S1. PRISMA NMA Checklist. Table S2. Search strategy. Table S3. Details of radiation fields. [file 13014_2019_1388_MOESM1_ESM.zip › Additional file 1 Fig. S2.tiff]

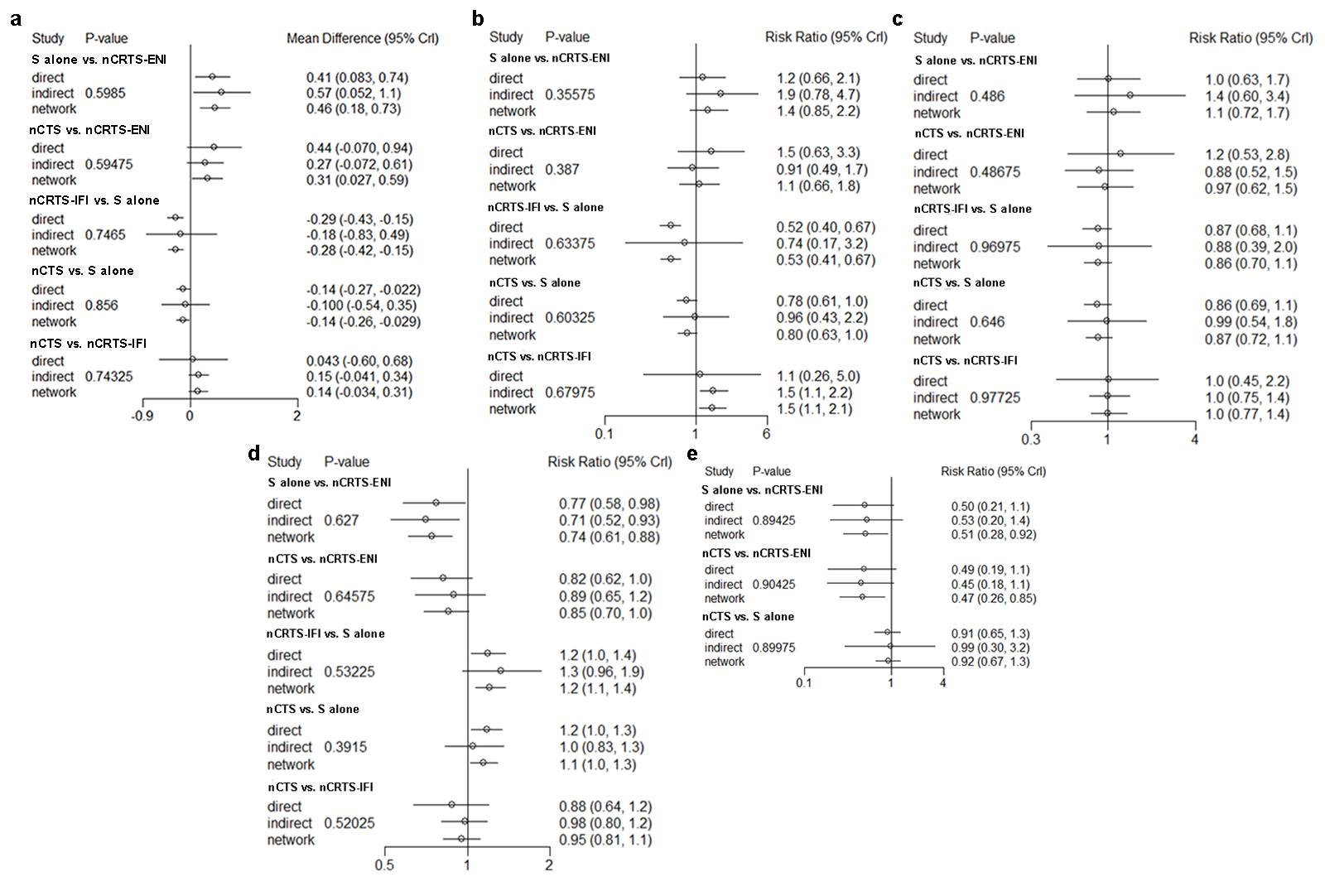

Supplement: Supplementary file 1 — Additional file 1: Figure S1. Assessment of risk of bias. A: Methodological quality graph: authors’ judgment about each methodological quality item presented as percentages across all included studies; B: Methodological quality summary: authors’ judgment about each methodological quality item for each included study, “+” low risk of bias; “?” unclear risk of bias; “-” high risk of bias. Figure S2. Comparison-adjusted funnel plots of publication bias test for overall survival. nCRTS, neoadjuvant chemoradiotherapy plus surgery; nCTS, neoadjuvant chemotherapy plus surgery; S, surgery; ENI, elective nodal irradiation; IFI, involved-field irradiation. Figure S3. Inconsistency evaluation by node-splitting analyses. (a) overall survival; (b) locoregional recurrence; (c) distant metastases; (d) R0 resection; (e) post-operative mortality. nCRTS, neoadjuvant chemoradiotherapy plus surgery; nCTS, neoadjuvant chemotherapy plus surgery; S, surgery; ENI, elective nodal irradiation; IFI, involved-field irradiation. Table S1. PRISMA NMA Checklist. Table S2. Search strategy. Table S3. Details of radiation fields. [file 13014_2019_1388_MOESM1_ESM.zip › Additional file 1 Fig. S3.tiff]
