# Supplementary material for: Elective nodal irradiation versus involved-field irradiation in patients with esophageal cancer receiving neoadjuvant chemoradiotherapy: a network meta-analysis
Source: Radiat Oncol. 2019 Oct 16;14:176. doi: 10.1186/s13014-019-1388-8 (PMC6794743; doi:10.1186/s13014-019-1388-8)
Supplement: Supplementary file 1 — Additional file 1: Figure S1. Assessment of risk of bias. A: Methodological quality graph: authors’ judgment about each methodological quality item presented as percentages across all included studies; B: Methodological quality summary: authors’ judgment about each methodological quality item for each included study, “+” low risk of bias; “?” unclear risk of bias; “-” high risk of bias. Figure S2. Comparison-adjusted funnel plots of publication bias test for overall survival. nCRTS, neoadjuvant chemoradiotherapy plus surgery; nCTS, neoadjuvant chemotherapy plus surgery; S, surgery; ENI, elective nodal irradiation; IFI, involved-field irradiation. Figure S3. Inconsistency evaluation by node-splitting analyses. (a) overall survival; (b) locoregional recurrence; (c) distant metastases; (d) R0 resection; (e) post-operative mortality. nCRTS, neoadjuvant chemoradiotherapy plus surgery; nCTS, neoadjuvant chemotherapy plus surgery; S, surgery; ENI, elective nodal irradiation; IFI, involved-field irradiation. Table S1. PRISMA NMA Checklist. Table S2. Search strategy. Table S3. Details of radiation fields. [file 13014_2019_1388_MOESM1_ESM.zip › Additional file 1 Tables S2.doc]

**Supplementary table S2** Search strategy

**a:** Search strategy in PubMed

| # | Query |
| --- | --- |
| #1 | “Esophageal Neoplasms”[mh] |
| #2 | Esophageal Neoplasm[tiab] OR Esophagus Neoplasm[tiab] OR Esophagus Cancer[tiab] OR Esophageal Cancer[tiab] OR Esophageal Cancers[tiab] OR Gastro-esophageal Junction Neoplasms[tiab] OR Esophagogastric Junction Neoplasms[tiab] |
| #3 | Esophageal[tiab] OR Oesophageal[tiab] OR Esophagus[tiab] OR Esophag*[tiab] OR Oesophag*[tiab] |
| #4 | Cancer*[tiab] OR Tumour*[tiab] OR Tumor[tiab] OR Neoplasm*[tiab] OR Carcinoma[tiab] |
| #5 | #3 AND #4 |
| #6 | #1 OR #2 OR #5 |
| #7 | Preoperative Chemoradiotherapy[tiab] OR Neoadjuvant Chemoradiotherapy[tiab] OR Chemoradiotherapy Followed By Surgery[tiab] OR Preoperative Chemotherapy[tiab] OR Neoadjuvant Chemotherapy[tiab] OR Chemotherapy Followed By Surgery[tiab] |
| #8 | Surgery[tiab] OR Esophagectomy[tiab] |
| #9 | #7 AND #8 |
| #10 | #6 AND #9 |
| #11 | Randomized Controlled Tial[pt] |
| #12 | Controlled Cinical Trial[pt] |
| #13 | Randomized[tiab] |
| #14 | Placebo[tiab] |
| #15 | Randomly[tiab] |
| #16  #17 | Trial[tiab]  Drug Therapy[sh] |
| #18 | Groups[tiab] |
| #19 | #11 OR #12 OR #13 OR #14 OR #15 OR #16 OR #17 OR #18 |
| #20 | Animals[mh] |
| #21 | Humans[mh] |
| #22 | #20 NOT #21 |
| #23 | #19 NOT #22 |
| #24 | #10 AND #23 |

**b:** Search strategy in Embase

| # | Query |
| --- | --- |
| #1 | ‘esophagus tumor’/exp |
| #2 | ‘esophagus tumor’:ab,ti OR ‘esophageal neoplasm’:ab,ti OR ‘esophagus neoplasm’:ab,ti OR ‘esophagus cancer’:ab,ti OR ‘esophageal cancer’:ab,ti OR ‘esophageal cancers’:ab,ti OR ‘gastro-esophageal junction neoplasms’:ab,ti OR ‘esophagogastric junction neoplasms’:ab,ti |
| #3 | ‘esophageal’:ab,ti OR ‘oesophageal’:ab,ti OR ‘esophagus’:ab,ti OR ‘esophag*’:ab,ti OR ‘oesophag*’:ab,ti |
| #4 | ‘cancer*’:ab,ti OR ‘tumour*’:ab,ti OR ‘tumor’:ab,ti OR ‘neoplasm*’:ab,ti OR ‘carcinoma’:ab,ti |
| #5 | #3 AND #4 |
| #6 | #1 OR #2 OR #5 |
| #7 | ‘preoperative chemoradiotherapy’:ab,ti OR ‘neoadjuvant chemoradiotherapy’:ab,ti OR ‘chemoradiotherapy followed by surgery’:ab,ti OR ‘preoperative chemotherapy’:ab,ti OR ‘neoadjuvant chemotherapy’:ab,ti OR ‘chemotherapy followed by surgery’:ab,ti |
| #8 | ‘surgery’:ab,ti OR ‘esophagectomy’:ab,ti |
| #9 | #7 AND #8 |
| #10 | 'trial':ab,ti |
| #11 | 'random*':ab,ti |
| #12 | 'clinical trial'/de OR 'controlled clinical trial'/de OR 'randomized controlled trial'/de |
| #13 | #10 OR #11 OR #12 |
| #14 | #6 AND # 9 AND #13 |

**c:** Search strategy in Cochrane Library

| # | Query |
| --- | --- |
| #1 | MeSH descriptor: [Esophageal Neoplasms] explode all trees |
| #2 | (esophageal neoplasm OR esophagus neoplasm OR esophagus cancer OR esophageal cancer OR esophageal cancers OR gastro-esophageal junction neoplasms OR esophagogastric junction neoplasms) |
| #3 | ((esophageal OR oesophageal OR esophagus OR esophag* OR oesophag*) AND (neoplas* OR cancer* OR carcinoma* OR tumour* or tumor)) |
| #4 | (preoperative chemoradiotherapy OR neoadjuvant chemoradiotherapy OR chemoradiotherapy followed by surgery OR preoperative chemotherapy OR neoadjuvant chemotherapy OR chemotherapy followed by surgery) |
| #5 | (surgery OR esophagectomy) |
| #6 | # 4 AND #5 |
| #7 | #1 OR #2 OR #3 |
| #8 | #7 AND #6 |

**d:** Search strategy in Web of Science

| # | Query |
| --- | --- |
| #1 | TI=("esophageal neoplasm” OR “esophagus neoplasm” OR “esophagus cancer” OR “esophageal cancer” OR “esophageal cancers” OR “gastro-esophageal junction neoplasms” OR “esophagogastric junction neoplasms” OR ((esophageal OR oesophageal OR esophagus OR esophag* OR oesophag*) AND (neoplas* OR cancer* OR carcinoma* OR tumour* or tumor))) |
| #2 | TI=("preoperative chemoradiotherapy” OR “neoadjuvant chemoradiotherapy” OR “chemoradiotherapy followed by surgery” OR “preoperative chemotherapy” OR “neoadjuvant chemotherapy” OR “chemotherapy followed by surgery”) |
| #3 | TI=(“surgery” OR “esophagectomy”) |
| #4 | #2 AND #3 |
| #5 | #1 AND #4 |
