# Supplementary material for: Elective nodal irradiation versus involved-field irradiation in patients with esophageal cancer receiving neoadjuvant chemoradiotherapy: a network meta-analysis
Source: Radiat Oncol. 2019 Oct 16;14:176. doi: 10.1186/s13014-019-1388-8 (PMC6794743; doi:10.1186/s13014-019-1388-8)
Supplement: Supplementary file 1 — Additional file 1: Figure S1. Assessment of risk of bias. A: Methodological quality graph: authors’ judgment about each methodological quality item presented as percentages across all included studies; B: Methodological quality summary: authors’ judgment about each methodological quality item for each included study, “+” low risk of bias; “?” unclear risk of bias; “-” high risk of bias. Figure S2. Comparison-adjusted funnel plots of publication bias test for overall survival. nCRTS, neoadjuvant chemoradiotherapy plus surgery; nCTS, neoadjuvant chemotherapy plus surgery; S, surgery; ENI, elective nodal irradiation; IFI, involved-field irradiation. Figure S3. Inconsistency evaluation by node-splitting analyses. (a) overall survival; (b) locoregional recurrence; (c) distant metastases; (d) R0 resection; (e) post-operative mortality. nCRTS, neoadjuvant chemoradiotherapy plus surgery; nCTS, neoadjuvant chemotherapy plus surgery; S, surgery; ENI, elective nodal irradiation; IFI, involved-field irradiation. Table S1. PRISMA NMA Checklist. Table S2. Search strategy. Table S3. Details of radiation fields. [file 13014_2019_1388_MOESM1_ESM.zip › Additional file 1 Tables S3.doc]

**Supplementary Table S3.** Details of radiation fields.

| Trial | Treatment | Irradiation range |
| --- | --- | --- |
| Yanagi/2018[17] | nCRTS-ENI | CTV was delivered to the mediastinum and neck. |
| **C**ALGB9781/2008[18] | nCRTS-ENI | CTV was defined as GTV of the involved field plus elective nodal region, with 5 cm beyond the |
|  |  | proximal and distal , and 2 cm lateral margin |
| Natsugoe/2006[19] | nCRTS-ENI | CTV was delivered to the mediastinum and neck. |
| Lee/2004[20] | nCRTS-ENI | CTV included the GTV of the involved field plus elective nodal region, with a 7 cm margin superior |
|  |  | and inferior and a 2 cm laterally |
| Le Prise/1994[21] | nCRTS-ENI | CTV was delivered to the mediastinum and the supraclavicular nodes |
| Nygaard/1992[22] | nCRTS-ENI | CTV was defined as the whole esophagus and mediastinum from the suprasternal notch |
|  |  | to the cardia |
| Stahl/2009[35-36] | nCRTS-ENI | CTV was defined as GTV plus 5 cm in the oral and a 3 cm aboral margin and the areas at risk for |
|  |  | elective nodal regions |
| Burmeister/2011[37] | nCRTS-IFI | CTV was defined as involved lymph nodes |
| NEOCRTEC5010/2018[5] | nCRTS-IFI | CTV was defined as enlarged lymph nodes, with a 3 cm proximal and distal margin and a 0.5-1.0 |
|  |  | cm radial margin |
| CROSS/2011[6-7] | nCRTS-IFI | CTV was defined as any enlarged regional lymph nodes |
| Lv/2010[8] | nCRTS-IFI | CTV was defined as enlarged lymph nodes, with a 4-5 cm proximal and distal margin and a 1-2 cm |
|  |  | radial margin |
| FFCD9901/2014[9] | nCRTS-IFI | CTV was defined as involved lymph nodes , and CTV extended to 3cm of mediastinal tissue |
|  |  | above and below the GTV |
| IG9401/2005[10] | nCRTS-IFI | CTV was defined as the GTV plus a 5 cm margin superior and inferior and a 3 cm laterally |
| Urba/2001[11] | nCRTS-IFI | CTV was defined as the GTV plus a 5 cm cephalo-caudad margin and a 2 cm radial margin |
| Bosset/1997[12] | nCRTS-IFI | CTV was defined as the GTV plus a 5 cm proximal and distal margin and a 2 cm radial margin |
| Walsh/1996[13-14] | nCRTS-IFI | CTV was defined as the GTV plus a 5 cm longitudinal margin and a 2-3 cm radial margin |
| Apinop/1994[15] | nCRTS-IFI | CTV was defined as GTV plus a 2 cm margin superior and a 5 cm inferior to the primary tumor. |
| Cao/2009[16] | nCRTS-IFI | CTV was designed to include enlarged regional nodes |

Abbreviations: nCRTS, neoadjuvant chemoradiotherapy plus surgery; ENI, elective nodal irradiation; IFI, involved-field irradiation; CTV, clinical target volume; GTV, Gross Tumor Volume.
